# Biosynthesis of Polyhydroxyalkanoates (PHAs) by the Valorization of Biomass and Synthetic Waste

**DOI:** 10.3390/molecules25235539

**Published:** 2020-11-26

**Authors:** Hadiqa Javaid, Ali Nawaz, Naveeda Riaz, Hamid Mukhtar, Ikram-Ul-Haq, Kanita Ahmed Shah, Hooria Khan, Syeda Michelle Naqvi, Sheeba Shakoor, Aamir Rasool, Kaleem Ullah, Robina Manzoor, Imdad Kaleem, Ghulam Murtaza

**Affiliations:** 1 Institute of Industrial Biotechnology (IIB), Government College University, Lahore 54000, Pakistan; javaid.hadiqa94@gmail.com (H.J.); ali.nawaz@gcu.edu.pk (A.N.); hamidmukhtar@gcu.edu.pk (H.M.); dr.ikramulhaq@gcu.edu.pk (I.-U.-H.); kanita.a.shah@gmail.com (K.A.S.); mnaqvi678@gmail.com (S.M.N.); sheebashakoor03@gmail.com (S.S.); 2 Department of Biological Sciences, International Islamic University, Islamabad 45550, Pakistan; naveeda.riaz@iiu.edu.pk; 3 Department of Biosciences, COMSATS University Islamabad (CUI), Islamabad 45550, Pakistan; hooriakhan.pk@gmail.com; 4 Institute of Biochemistry, University of Balochistan, Quetta 87300, Pakistan; rasool.amir@gmail.com; 5 Department of Microbiology, University of Balochistan, Quetta 87300, Pakistan; drkaleemullah@gmail.com; 6 Faculty of Marine Sciences, Lasbella University of Agriculture, Water and Marine Sciences, Balochistan 90150, Pakistan; 3820150009@bit.edu.cn; 7 Department of Zoology, University of Gujrat, Gujrat 50700, Pakistan; gmurtazay@yahoo.com

**Keywords:** biomass valorization, biodegradation, biopolymer, biomaterials, bioplastic, plastic bag, biological materials, microbial polymers, bacterial bioplastic, eco-friendly materials

## Abstract

Synthetic pollutants are a looming threat to the entire ecosystem, including wildlife, the environment, and human health. Polyhydroxyalkanoates (PHAs) are natural biodegradable microbial polymers with a promising potential to replace synthetic plastics. This research is focused on devising a sustainable approach to produce PHAs by a new microbial strain using untreated synthetic plastics and lignocellulosic biomass. For experiments, 47 soil samples and 18 effluent samples were collected from various areas of Punjab, Pakistan. The samples were primarily screened for PHA detection on agar medium containing Nile blue A stain. The PHA positive bacterial isolates showed prominent orange–yellow fluorescence on irradiation with UV light. They were further screened for PHA estimation by submerged fermentation in the culture broth. Bacterial isolate 16a produced maximum PHA and was identified by 16S rRNA sequencing. It was identified as *Stenotrophomonas maltophilia* HA-16 (MN240936), reported first time for PHA production. Basic fermentation parameters, such as incubation time, temperature, and pH were optimized for PHA production. Wood chips, cardboard cutouts, plastic bottle cutouts, shredded polystyrene cups, and plastic bags were optimized as alternative sustainable carbon sources for the production of PHAs. A vital finding of this study was the yield obtained by using plastic bags, i.e., 68.24 ± 0.27%. The effective use of plastic and lignocellulosic waste in the cultivation medium for the microbial production of PHA by a novel bacterial strain is discussed in the current study.

## 1. Introduction

Plastics and pollution are two deeply connected terms. The plastic industry is an ever-growing entity expected to reach a value of nearly $2184.26 billion by 2022 [1]. Pakistan’s plastic industry is growing at an annual growth rate of 15% with 624,200 million tons/per annum estimated production rate. A value investment of about $260 billion was attracted by this industry among which half of the investments were foreign direct investments (FDI). These foreign investments contribute to 35% of the outstanding export growth of plastics in Pakistan [2]. Plastics have invaded almost all areas of our daily lives; from transportation, electronics, medical industry, textiles to packaging, etc. [3]. Depending on the plastic product, it can take approximately 600 years to degrade [4]. Plastic overuse has resulted in depleting fossil fuels, severely endangered wildlife, impacts on human health, global warming, solid waste accumulation, hazardous air, and water pollution. Plastic pollution has become such a widespread phenomenon that our environment is now home to 51 trillion pieces of plastics. This figure is 500 times more than the number of stars in our galaxy [5]. In the next 20 years, plastic pollution is expected to double putting our current waste management and recycling practices to a shame [6]. The latest research suggests that the presence of macro and micro-plastics in the environment is such immense that it is factually “raining plastics” [7,8,9].

For the past two decades, “Bioplastics” or “Bio-based polymers” have emerged as prominent keywords. Renewable materials, such as starch, cellulose, plant oils, chitin, pectin, soy protein, whey protein, collagen, and gelatin are important sources for bioplastic production. Polylactic acid (PLA) and polyhydroxyalkanoates (PHAs) represent the two most promising and the most important bioplastics of the modern age produced by plants, algae, and bacteria using renewable sources [10]. Over 300 heterotrophic gram-negative and gram-positive bacterial species capable of synthesizing PHAs have been isolated and identified e.g., *Methylobacterium* sp., *Cupriavidus necator*, *Bacillus* sp., *Pseudomonas* sp., *Enterobacter* sp., *Citrobacter* sp., *Escherichia* sp., *Klebsiella* sp., *Azotobacter beijerinckii*, *Rhizobium* sp., *A. vinelandii*, *A. macrocytogenes*, *C. necator*, *P. oleovorans*, and *Protomonas extorquens*, etc. [11]. The search for novel bacterial species carrying enhanced potential of PHA production has resulted in the discovery of such potential bacterial strains that produce PHAs efficiently. Some recent novel strains of *Stenotrophomonas* sp., *Xanthomonas* sp., *Staphylococcus* sp. and Haloarchaea bacteria [12] are a few more examples of such bacteria [13].

PHAs are made up of various hydroxycarboxylic acid polyesters, which are produced by a large number of bacteria. They are hydrophobic inclusions formed in bacterial cells in the excess of carbon and limitation of other nutrients, such as N, O, P, S or Mg. They are used as reserves of carbon and energy [14]. They are eco-friendly, 100% biodegradable, recyclable, non-toxic, biocompatible, and bioresorbable [15]. When buried in soil, PHAs decompose completely into carbon dioxide and water within seven months [16]. However, the cost of inoculum preparation, downstream processing, and raw materials makes its commercial preparation 5–10 times expensive than normal plastics [17]. Carbon sources cost more than 50% of the process. Therefore, the trend is shifting towards the use of cheap, waste, and sustainable substrates, which are easily processed or need no processing at all before use [18]. Most experts in the field are of the view that each step in the industrialization of PHAs still requires extensive research, optimization, and understanding to make it more sustainable in practice [19].

Since plastics are mostly based on carbon atoms, they can be an excellent carbon source to produce PHAs [20]. Plastics have many types but the most common types are polyethylene terephthalate (PET), polyvinyl chloride (PVC), foamed polystyrene and polymethylmethacrylate (Plexiglas). On the basis of chemical composition, plastics are of two types. One type is entirely made of linear (aliphatic) carbon atoms in the backbone. Other category includes heterochains, such as N, O, or S atoms in the structure other than C [21]. There are many concerns involved with using plastics in these processes. Nevertheless, with the appropriate alliance in value chains, this approach can prove fruitful in plastic waste management.

Considering the current research on sustainable carbon sources to produce bioplastics, the bioplastic industry will potentially boom in the near future. The global bioplastic production capacity is set to increase from around 2.11 million tons in 2018 to approximately 2.62 million tons in 2023, with PHAs being the main drivers of the market [22]. This research focused on the isolation of a new PHA producing bacterial strain from soil and effluent samples to be used in developing a cost-effective method for the synthesis of PHAs by synthetic waste and lignocellulosic biomass as substrates.

## 2. Results and Discussion

### 2.1. Primary Screening

**Figure 1:**
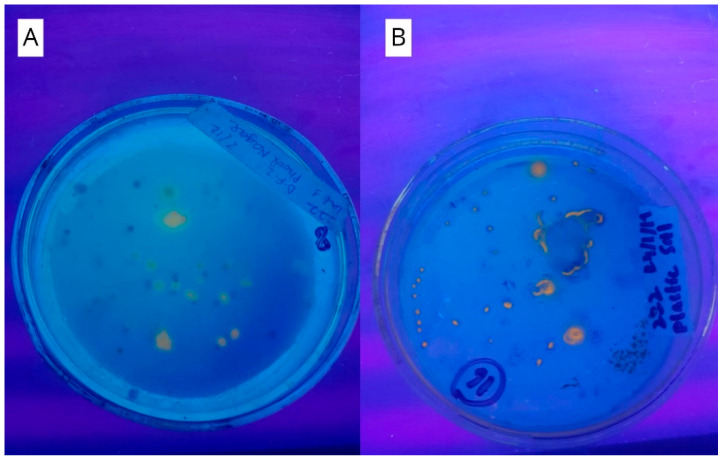
PHA producing samples inoculated on PHA detecting agar, exhibiting fluorescence under UV light. (**A**) Shows a petri plate of sample 8 and (**B**) shows growth on the petri plate by sample 16.

A total of 65 samples, including 47 soil and 18 effluent samples were collected from various areas of Punjab, Pakistan. These samples were subjected to primary screening for the isolation of PHA producing bacteria. A total of 127 bacterial isolates were screened from these samples. Moreover, 67 isolates revealed signs of PHA accumulation by showing orange–yellow fluorescence under UV light as mentioned in Table 1. Consequently, these 67 isolates were further selected for the estimation of PHA production. Figure 1A shows an example of a PHA positive sample (sample no: 8).

PHAs are insoluble storage granules that microorganisms accumulate in stressful environmental conditions, under excess of carbon and deficiency of other essential nutrients [23]. Thus, industrial soil samples and effluents used for the isolation of PHA producing bacteria were appropriate for the purpose. Most of the samples were collected from the industries of paint, paper, and plastic.

Any substance that becomes useless and defective after its primary use is considered as “waste” [24]. Liquid waste from industrial sites, agricultural processes or domestic sewage is “effluent waste”. Effluents can be harmful to the environment if released untreated because of their polluting chemical nature [25], as they contain partially degraded organic matter with minimum nutrients. They serve as an ideal source of isolation for microorganisms adapted to survive in oligotrophic conditions [26]. Products difficult to degrade such as plastic, paint residues, cardboard, and paper residues may also be a source of isolation of novel microorganisms that degrade these difficult products with simultaneous PHA production. Whereas soil samples collected from agricultural areas, such as croplands, compost, and landfill sites are rich in carbon sources [27]. The choice of collecting the samples from these habitats has made possible the isolation of a large number of PHA positive isolates.

For a more visual detection of PHA producing bacteria, staining and fluorescence come into play. This is achieved through the use of Nile blue A stain in the agar medium. When the cells of some microbial colonies grow in a medium containing this stain, the stain is absorbed within the cytoplasm of their cells [28]. This dye subsequently enters into the PHA inclusions. It is a basic oxazine dye [29] containing Nile blue sulfate and Basic Blue 12. These compounds when excited by UV light of 312 nm, reflect orange color hence the orange fluorescence is generated [28]. Apart from being a highly sensitive method to detect PHAs, it also indicates the difference in the amount of accumulated PHAs [29]. The greater the intensity of fluorescence, the more the accumulation of PHA granules [30]. In this way, the isolate 16a showed the brightest and the most intense fluorescence as seen in Figure 1B. It was concluded that this strain held the highest potential to produce maximum PHA content as compared to other strains.

### 2.2. Secondary Screening of PHA Producers

Primarily screened bacterial colonies were further purified by quadrant streaking and analyzed for PHA production by submerged fermentation at 37 °C, 150 rpm for 72 h. After incubation, maximum PHA production was shown by the isolate 16a i.e., 69.72 ± 0.17%. However, isolates 3a, 4a, 5a, 8a, 11a, 12a, 14a, 16b, 18b, 23a, 33a, 39a, 45a, 47a, 48a, 53a, and 65a showed significant PHA production ranging from 36% to 55%, approximately. While the remaining strains did not show any significant PHA production, as evident from Table 2. Bacterial isolate 16a was selected for further studies.

Since the isolate 16a produced maximum PHA, it could be quite possible that this strain could quickly and efficiently utilize carbon source. The source of isolate 16a was plastic industry soil. Mohammed et al. [30], Kosseva and Rusbandi [31], and Sangakharak and Prasertsan [32] reported the isolation of bacteria from similar plastic sources, such as plastic pieces, plastic chairs, and plastic waste landfill sites, which provided high PHA yields up to 0.5 g/100 mL. High PHA production rate is associated with the bacterial ability to utilize plastic as a substrate as the soils enriched with plastic pieces are their indigenous habitats. These strains already have modified metabolism rates to sustain in oligotrophic conditions so they adapt to utilize the carbon available in plastic [33]. This shows an enhanced ability of indigenous bacteria to survive in nutrient-deficient conditions than non-indigenous bacteria [34]. In this research, the outcome of considerable differences in the PHA yields of bacteria isolated from plastic habitats can be due to the differences in the surrounding habitats of the plastic sources. Puglisi et al. [35] studied and proved the hypothesis that different polyethylene (PE) plastic waste samples harbor different bacterial communities. The structure and physiological capabilities of these communities are dependent on the physico-chemical properties of the plastic waste and the environment in which they dwell.

### 2.3. Molecular Identification

Sequencing of isolate 16a was carried out by Macrogen sequencing company, Seoul, Korea. Primers used for PCR and 16S rRNA sequencing are given in (Table 3).

**Figure 2:**
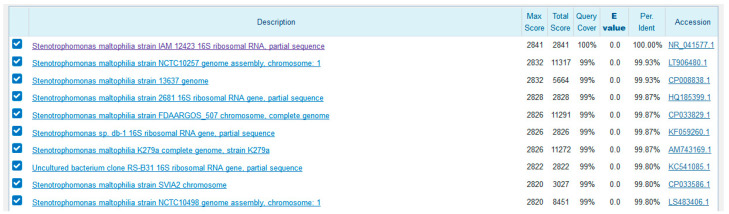
Nucleotide Basic Local Alignment Search Tool (BLAST) results for the isolated strain: 16a. The first ten homologues were selected for phylogenetic tree construction.

**Figure 3:**
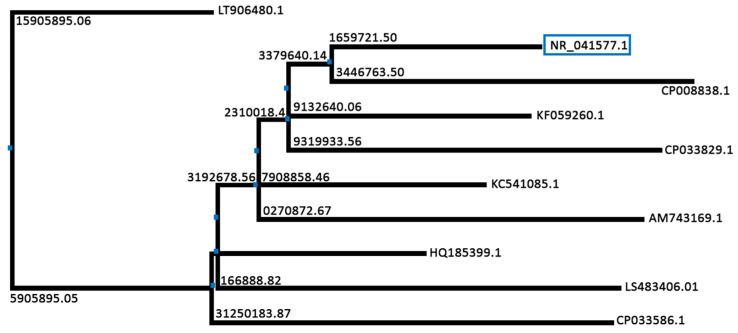
An illustration of the neighbor-joining phylogenetic tree of identified isolate 16a: *Stenotrophomonas maltophilia* strain IAM 12423. The numbers indicate the evolutionary distance, whereas the labels at the end of the arms represent the accession numbers of the BLAST homologues. The blue pointers indicate nodes.

The sequencing results were put into Basic Local Alignment Search Tool (BLAST) for homology analysis and first ten homologues were selected (Figure 2) for phylogenetic tree construction using the Jalview application (Figure 3) [36,37]. The results of the BLAST revealed that the gene sequence of isolate 16a is having identity with *Stenotrophomonas maltophilia* strain IAM 12423. Therefore, it was determined that the strain used in this research is *S. maltophilia*. GenBank sequence of the identified strain was submitted under the name of *Stenotrophomonas maltophilia* HA-16 with the accession number MN240936. The genus *Stenotrophomonas* is phylogenetically placed in the Gammaproteobacteria, was first described with the type species *Stenotrophomonas maltophilia* [38]. The *Stenotrophomonas* genus is a gram-negative genus with at least ten species [39]. It belongs to the family Xanthomonadaceae [40].

Horiike [41] dictates the importance of phylogenetic trees in molecular identification. He emphasizes that phylogenetic trees are much helpful in predicting the evolutionary basis of relationships between various species. These trees can help us predict the effects of evolutionary patterns in different habitats and their effects on a strain’s metabolic products. They also tell us about the evolution of metabolic products from ancestors to their descendants and how they are improved or differentiated over time [42].

### 2.4. Characterization of the Extracted Polymer

**Figure 4:**
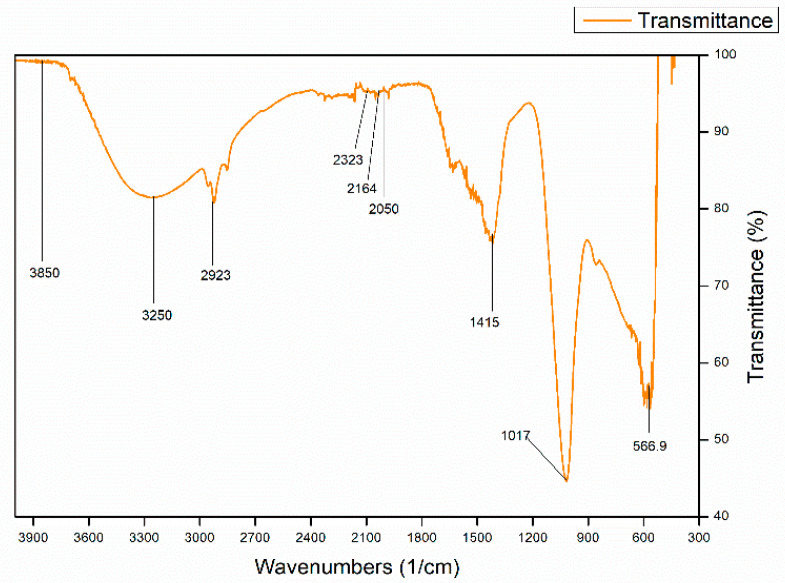
FTIR peaks for the extracted polymer within the transmittance range of 400–4000 cm^−1^. The labels indicate the peaks through which functional groups are analyzed and compared with the peaks of the standard polymer, in this case, PHB.

The PHA extracted from *S. maltophilia* HA-16 was characterized by FTIR spectroscopy. Functional group analysis was done through signal peaks recorded in the range of 4000–400 cm^−1^. The peak was plotted using OriginLab 8.5 as shown in Figure 4 [43].

Abid et al. [44] compared his extracted polymer with FTIR peaks of standard Polyhydroxybutyrate (PHB) purchased from Sigma Aldrich© (St. Louis, MO, USA). We compared our polymer with the FTIR peaks of the standard PHB sample used by Abid et al. [44]. The Fingerprinting region is usually the region of the spectrum in the range of 670–400 cm^−1^. The polymer under study expressed a considerable peak at 566.9 cm^−1^, which showed that there were many stretches of carbonyl groups (C=O) present. Comparatively, the fingerprinting region of the standard PHB had multiple sharp peaks, the sharpest one being at 514.03 cm^−1^. Absorption in the range of 1200–800 cm^−1^ indicated the presence of multiple C-C stretches with medium length bands which corresponded to an alkane group [45]. The extracted polymer expressed a sharp peak at 1017 cm^−1^, whereas the standard showed a sharp peak at 1044.67 cm^−1^. The sharpness of the peaks in both the polymers was almost the same which signified the same length of C-C stretches.

Pérez-Arauz et al. [46] mentioned that absorption peaks in the range of 3000–2850 cm^−1^ show a strong C-H bond stretch. The extracted polymer displayed a small peak at 2923 cm^−1^ in comparison to the standard which expressed a same sized peak at 2933.36 cm^−1^. Since the C-O peaks are not very prominent in the main region, this difference could be due to the disturbances in the polymer structure occurred during extraction [47]. The comparison of the size and the location of peaks in between the standard PHB and our extracted polymer showed that our polymer might not be as pure. The presence of similar length patterns in the peaks at some places can indicate that the biosynthesized polymer is a medium chain length (mcl) PHA copolyester [48].

### 2.5. Optimization of Cultural Conditions

Incubation time, incubation temperature, pH of the fermentation medium, and carbon sources were optimized for maximum PHA production through duplicate fermentation experiments. The average percentage amount of PHA produced with intracellular cell dry weight (CDW g/100 mL), per experiment per parameter is expressed in Table 4.

#### 2.5.1. Effect of Time of Incubation

**Figure 5:**
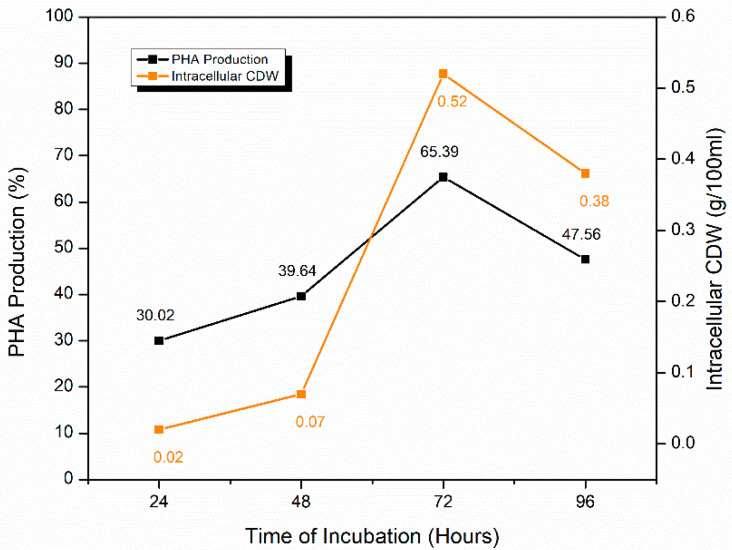
Optimization of time of incubation for PHA production by *S. maltophilia* HA-16 with glucose as a carbon source.

Effect of incubation time (24, 48, 72, and 96 h) was studied with glucose as a carbon source for PHA production. PHA production was observed to increase from 24 h and kept on increasing for 72 h where maximum PHA production (65.39 ± 0.42%) was expressed. Contrarily, at 96 h, PHA production declined to 47.56 ± 0.37%. As a result, 72 h incubation time was optimized for further experiments (Figure 5). PHA percentage seems to be directly related to the amount of intracellular CDW (g/100 mL). Nonetheless, at 72 h incubation, 0.52 g/100 mL of intracellular CDW was extracted which was recorded the highest in this pool of experiments.

Contrarily, Munir et al. [49] expressed a different trend for PHA production by *Stenotrophomonas* genus with highest PHA yield achieved after 48 h. They used 2% glucose as the sole carbon source compared to our study where only 0.45% of glucose was used. They recorded increasing growth until 72 h, but PHA production increased until 48 h, only and after that, it started declining. Additionally, this difference can be backed by the work of Alqahtani [50], where she describes 48 h as the optima for the highest metabolic activity for PHA production. Shaaban and Mowafy [51] described that the maximum PHA production by *S. maltophilia* occurred at 96 h and stayed stable until 144 h. They utilized 1% glucose in the medium. These differences in the optimum incubation time to produce maximum PHA might be attributed to differences in the nutrients and the carbon source (which in our case was glucose).

#### 2.5.2. Effect of Temperature of Incubation

**Figure 6:**
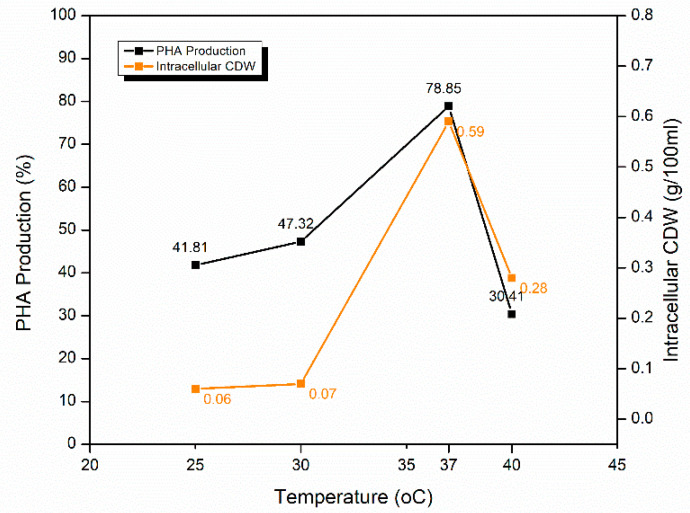
Optimization of the temperature of incubation for PHA production by *S. maltophilia* HA-16 with glucose as a carbon source.

PHA production was optimized at 25, 37, 30, and 40 °C with glucose as a carbon source to find out the temperature optima for *S. maltophilia* HA-16.37 °C was the optimum temperature with the highest PHA production i.e., 78.85 ± 0.23% as compared to other temperatures (Figure 6). At the same temperature, intracellular CDW was calculated to be the highest i.e., 0.59 g/100 mL. PHA content was low at 25 °C with a slight increase at 30 °C and a sharp decline after 37 °C with the lowest PHA production at 40 °C. PHA production at 25 and 30 °C can be considered as satisfactory with 41.81 ± 0.39 and 47.32 ± 0.12%, respectively. However, PHA yield poorly declined at temperatures above 37 °C, producing only 30.41 ± 0.47% PHA at 40 °C. A strange observation in this experiment was recorded in the intracellular CDW at this temperature. Usually, CDW is observed to be directly related to the amount of PHA produced. Yet, CDW at 40 °C was recorded to be 0.2 g/100 mL, which was still higher than the CDW recorded at 25 °C and 30 °C. Hence, it cannot be taken as a thumb rule that the higher the CDW, the higher the PHA content, because the CDW can also include the dry weight of things other than PHA.

Temperature optimization is important concerning the microorganism used for PHA production [52]. Scientists at the American Tissue Culture Center (ATCC) also confirmed 37 °C as the temperature optima for *S. maltophilia*. On the other hand, Singh and Parmar [13] reported PHA production with the same bacteria but different strains (*S. maltophilia* AK21 and *S. maltophilia* 13635L) at considerably low temperatures of 25 and 30 °C. This deviation might be due to the isolation of bacterial strains from different habitats. Alqahtani [50], in her work, demonstrated that extremely warm (55 °C) and extremely cold (4 °C) temperatures affect the growth of *S. maltophilia*. Her study further solidifies the results of this research by similar results, displaying the optimum growth at 37 °C. Guerrero and others [53] reported that PHA producing enzymes do not work efficiently above 37 °C and resulted in low yield.

#### 2.5.3. Effect of pH of the Fermentation Medium

**Figure 7:**
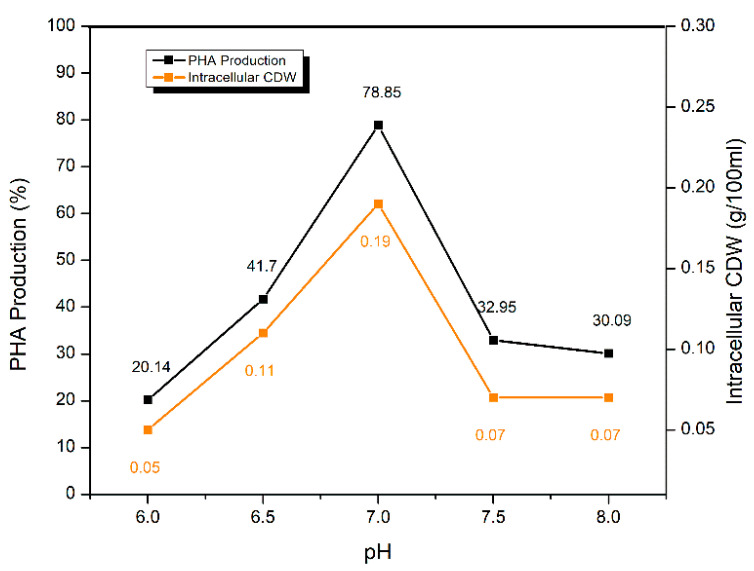
Optimization pH of fermentation medium for PHA production by *S. maltophilia* HA-16 with glucose as carbon source.

The fermentation medium was prepared at five different pH levels (6.0, 6.5, 7.0, 7.5, and 8.0) to find the pH optima for PHA production. A significant PHA yield of 78.85 ± 0.11% was recorded at 7.0 pH as compared to other pH levels. At acidic pH i.e., 6.0, PHA yield was poor i.e., only 20.14 ± 0.26% which increased to 41.70 ± 0.29% at slightly less acidic pH of 6.5. At slightly basic pH i.e., 7.5, 32.95 ± 0.33% of PHA production was observed. This yield is almost equal to the PHA yield recorded at 8.0 pH (Figure 7).

Shaaban with colleagues [54] found pH 7.0 as the optimum for PHB production by *S. maltophilia*. They further elaborated that at pH 6.0 and 8.0, PHB production was not significant which also related with the current study. Raj and his team [55] also reported pH 7.0 as the optimum pH for PHA production by *S. maltophilia*. Lathwal et al. [56] demonstrated the same results for PHA production at different pH of fermentation media. They were able to verify that PHA production was maximum at pH 7.0. Their work also validated the current results that on pH 6.0, PHA production was low, however, at pH 8.0, PHA production was not low and still significant.

#### 2.5.4. Optimization of Carbon Source for PHA Production

**Figure 8:**
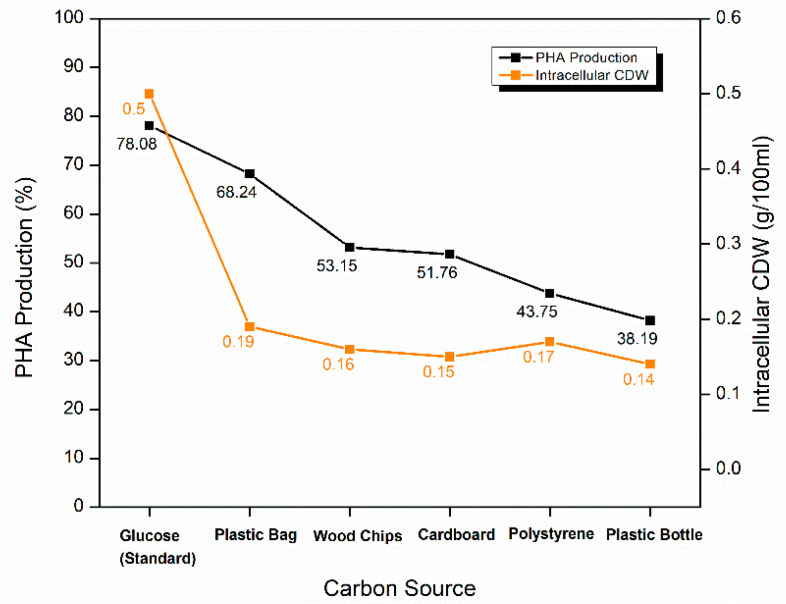
Optimization waste agro-industrial carbon sources for PHA production by *S. maltophilia* HA-16 with glucose as carbon source.

Some unconventional carbon sources such as undegraded wood chips, cardboard cutouts, shredded plastic bottles, wasted polystyrene cups, and waste plastic bags were used for increased PHA production. These carbon sources were used without any pre-treatments. Surprisingly, plastic bags proved the most optimum among these carbon sources and produced PHA content of 68.24 ± 0.27%. Whereas the other two plastic carbon sources (shredded polystyrene cups and plastic bottle cutouts) did not prove to be much efficient in PHA production. They produced the lowest PHA content of 43.75 ± 0.30% and 38.19 ± 0.22%, respectively. PHA content extracted from the wood chips and cardboard was almost equal i.e., 53.15 ± 0.17% and 51.76 ± 0.48%, respectively (Figure 8).

The cost of carbon sources is one of the prime difficulties in PHA commercialization as these sources contribute to more than 50% of the total industrial production costs. Glucose, among the optimized carbon sources, proved to be the best carbon source for PHA production, as also confirmed by Singh and Parmar [13]. However, the use of industrially produced glucose adds to the costs considerably. Replacing glucose in the fermentation medium with waste sources in the current research was an attempt to address this concern. When the same approach will be industrially adapted, the costs can be further cut down by integrating the production lines with waste streams of paper industries, plastic industries and packaging industries. However, the collection of plastic at the end of PHA production cycle does not ensure its complete breakdown. There are still concerns that need more attention. Since the use of plastics to produce PHAs is a new approach, extensive research to study all related industrial parameters are needed to make it a reality. Jimenez’s team [57] found *S. maltophilia* associated with the gut of Bark Beetle *Dendroctonus rhizophagus* (Curculionidae: Scolytinae). In its gut, it plays a role in the degradation, hydrolysis, fermentation, and oxidation of lignin and cellulose derived aromatic products. Their findings can elaborate the current results of PHA production by *S. maltophilia* HA-16 (MN240936) through wood chips and cardboard cutouts. In another study by Kirtania et al. [58], it was found that *S. maltophilia* has a significant ability to naturally degrade cellulose and hemicellulosic materials. Furthermore, Ali Wala’a et al. [59] reported numerous pretreated cellulosic and lignocellulosic sources giving maximum PHA production yield up to 90% which does not align with this research. In the current study, cellulosic and lignocellulosic materials gave lower yields as compared to one synthetic source i.e., plastic bag.

Plastics are of various types. One of the most abundant types of plastics is polyethylene terephthalate (PET). PET plastics are made of repeating units of polymer ethylene terephthalate [60]. Plastic bags are another very common plastic products which are made up of low density poly ethylene (LDPE) and/or high density poly ethylene (HDPE) [61]. The reason why PET and most plastics do not easily biodegrade is because the entire plastic structure has very strong C-C bonds that require too much energy to breakdown and plastics do not dissolve in water [62]. However, with the increased plastic accumulation in our environment, microbial life forms have evolved to degrade plastic products to some extent. There are many bacteria that have the ability to degrade PET, Polyethylene (PE), and Polystyrene (PS) but their enzymes only have been able to give moderate turnouts. The enzymes involved in PET degradation are known as PET hydrolases [20].

While working with synthetic plastics, Kenny and his research team [63] used pre-degraded PET as a carbon source and received a yield of only up to 21% with *Pseudomonas frederiksbergensis* GO23. Whereas, *S. maltophilia* HA-16 (MN240936) produced up to 38% of PHA with undegraded PET bottles. In another study, Dai and Reusch [64] utilized synthetic plastics by using the pyrolysis oil of polystyrene and reported PHA production up to 48% in tryptic soy broth (TSB) medium with *Cupriavidus necator* H16. Contrarily, *S. maltophilia* HA-16 gave a yield of 43% with undegraded or pretreated polystyrene fragments, which is still impressive. These yields indicate an evident ability of *S. maltophilia* HA-16 to degrade PET bottles and polystyrene. Current research is the first case of PHA production reported from the strain *S. maltophilia* HA-16 (MN240936).

A surprising finding of this study is the high yield of PHA, i.e., 68.24% in plastic bags. *S. maltophilia* is a gram-negative, non-fermentative bacterium that is present ubiquitously in various anthropogenic and natural environmental habitats [36]. It is a frequent colonizer of the rhizosphere and, hence, this species is present in various types of soils worldwide [65]. *S. maltophilia* has also been found as a part of the natural microbiome of various amoebal genera that are free-living [66]. *S. maltophilia* holds bioremediation capability of sites polluted with hydrocarbons and various xenobiotics [67]. What is more surprising about *S. maltophilia* is that, not only it is a potential PHA producer, but it is also a potential PHB degrader, as demonstrated by Wani et al. [68]. This bacterium is kind of like hitting two targets with one bullet, where it degrades its creation as well. It can immensely increase recycling in PHA production processes, hence, promoting a circular economy. The use of plastic bags as a carbon source for PHA production by any bacteria is yet unreported. This study could be the first of its kind to report such impressive PHA yields by the undegraded plastic bag as a carbon source, opening up new horizons in the field of plastic bag biodegradation and bioconversion by *S. maltophilia* HA-16 (MN240936). However, their effectiveness still needs to be improved with the aid of genetic engineering [69].

### 2.6. PHA Film Preparation

PHA polymer film was prepared by adding chloroform into the extracted polymer and evaporating it at 60 °C. The film obtained after preparation with chloroform was somewhat in the shape of fragments as compared to the quality of standard PHB film. Its durability still needs further improvement.

**Figure 9:**
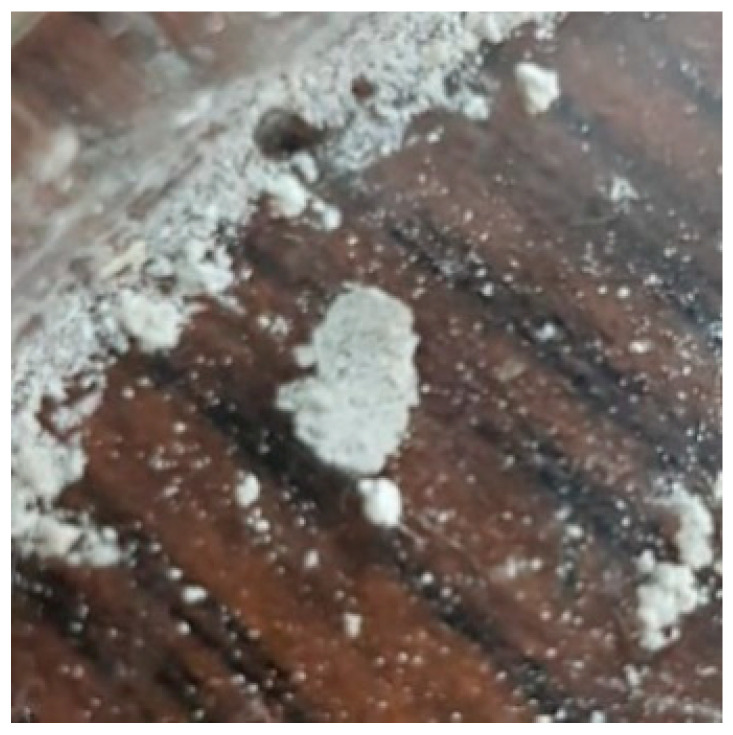
Brittle and Fragile film of extracted polymer.

The film obtained after drying with chloroform was very brittle and fragile (Figure 9). Mohammed et al. [30] reported a similar kind of brittle film made entirely of PHB polymers. He further added that these films are delicate unless made in combination with copolymers. Our lab-scale experiment was carried out with minimum resources so the copolymerization to achieve a proper film out of the extracted polymer was not possible.

## 3. Materials and Methods

### 3.1. Reagents Preparation

#### 3.1.1. Trace Elements Solutions

Trace elements solution (500 mL) was prepared by weighing 5 g of FeSO_4_·7H_2_O, 1.1245 g of ZnSO_4_·7H_2_O, 0.5 g of CuSO_4_·5H_2_O, 0.25 g of MnSO_4_·5H_2_O, 1 g of CaCl_2_·2H_2_O, 0.115 g of Na_2_B_4_O_7_·10H_2_O and 0.05 g of (NH)_4_MO_7_O_24_ using electric weight balance (Type AUW 2200 No. D 450013067, SHIMADZU Corporation Japan) and mixing them in 100 mL of distilled water and the final volume was raised to 500 mL [42].

#### 3.1.2. HCl (=35%) Solution Preparation

HCl (=35%) solution was prepared by adding 35 mL of concentrated HCl in 50 mL of distilled water and the volume was raised to 100 mL by distilled water.

### 3.2. Media Preparation

#### 3.2.1. PHA Detecting Agar

PHA detecting agar (500 mL) was prepared by adding 4 g of nutrient broth, 10 g of nutrient agar, and 0.25 mg of Nile blue A dye in 100 mL of distilled water, and the final volume was raised to 500 mL with distilled water. The media was sterilized in the autoclave (Model: WAC-60, Wisd, WiseStri, Germany) at 121 °C, 15 psi for 20 min. After sterilization, the media was poured aseptically in petri plates, which were pre-sterilized in Digital Oven (SNB-100, hot air sterilizer, Memmert) and stored in a Varioline Intercool cold cabinet at 4 °C until further use [70,71].

#### 3.2.2. Nutrient Broth and Nutrient Agar

The nutrient broth was prepared by dissolving 0.4 g of nutrient broth in 20 mL of distilled water and the final volume was raised to 50 mL with distilled water. The broth was sterilized by autoclaving at 121 °C, 15 psi for 20 min [71].

Nutrient agar (100 mL) was prepared by adding 0.8 g of nutrient broth and 1.5 g of agar in 50 mL of distilled water and was homogenized. The final volume of the medium was raised to 100 mL by distilled water. The medium was autoclaved at 121 °C, 15 psi for 20 min [72].

#### 3.2.3. Fermentation Medium

The fermentation medium was prepared by adding 0.318 g of Na_2_HPO_4_, 0.135 g of KH_2_PO_4_, 0.235 g of (NH_4_)_2_SO_4_, 0.0195 g of MgSO_4_, 0.05 g of nutrient broth and 0.45 g of pre autoclaved solution of glucose in 10 mL of distilled water. The volume was raised to 50 mL by adding sterile distilled water. Separately autoclaved solution of trace elements was also added in the medium in a concentration of 1 mL/L. The medium was autoclaved at 121 °C, 15 psi for only 5 min to prevent glucose from caramelization [73].

#### 3.2.4. Seed Culture Preparation

Twenty-four hours before fermentation, each colony was aseptically inoculated in 50 mL of autoclaved nutrient broth and incubated at 37 °C/150 rpm in a shaking incubator (Innova^®^ 43 Incubator Shaker Series) for 24 h to get an overnight old seed culture for the main fermentation process [74].

### 3.3. Sample Collection

Soil, compost, solid waste landfill soil, and industrial effluent samples were collected from paint and plastic industries, sugar mills, food, paper, pulp and cardboard industries, etc. Soil samples were collected aseptically in sterilized polythene bags while effluent samples were collected in sterilized plastic containers from Lahore, Gujrat, Mandi-bahauddin, Narowal, and other areas of the province Punjab, Pakistan [75]. Table 1 shows the geographical distribution of the areas from where the samples were collected.

### 3.4. Waste Collection

**Figure 10:**
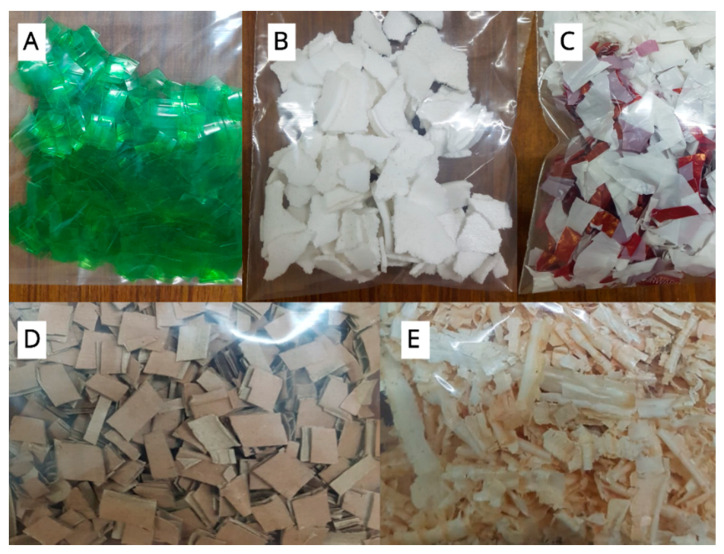
Photographs of carbon sources used for optimization of PHA production. (**A**) Plastic cutouts of a plastic bottle, (**B**) shredded waste polystyrene cups, (**C**) shredded plastic bag, (**D**) cardboard carton cutouts, (**E**) wood chips.

Wastes, such as wood chips, cardboard, plastic bottle, wasted polystyrene cups, and waste plastic bags were collected aseptically in sterilized polythene bags from local landfills and dumps of Lahore, Pakistan. The waste was used as a carbon source for the production of PHA [64]. Before inoculation, the waste was shredded into smaller pieces followed by sterilization under the UV hood [76] (Figure 10). Collected plastic bottles were made of PET (Polyethylene terephthalate). It’s an aliphatic polyester made of monomers obtained by either esterification of terephthalic acid with ethylene glycol or transesterification of ethylene glycol with dimethyl terephthalate [77]. The presence of a large aromatic ring in the PET repeating units gives the polymer notable stiffness and strength, especially when the polymer chains are aligned with one another in an orderly arrangement [78]. Thick solid colored plastic bags like the ones used in this study are linear HDPE polymers. PE is a polymer formed by radical polymerization of ethylene. Due to the linear structure of HDPEs, they are flexible, durable and tough [79]. Foamed PS used in this research commercially also known as Styrofoam© is a synthetic aromatic hydrocarbon polymer formed of styrene monomers. Its alternating C centers are attached to phenyl groups through σ bonds [80]. Styrofoam is a syndiotactic polymer, which has phenyl groups positioned to alternating sides of the C chain. This structure gives it a highly crystalline and hence a brittle structure [81].

### 3.5. Isolation and Screening

Qualitative isolation and screening of PHA producing bacteria was initially done by culturing the samples on PHA detecting agar containing Nile blue A stain by the method of Bhuwal et al. [76]. The plates after incubation were illuminated under UV light at 312 nm in UVP Mini Benchtop Transilluminator (Model: TM-10E) for the presence of bright orange fluorescence which indicates PHA accumulation in cells [75]. The initial screening experiments for each sample were run in duplicates. Colonies with orange fluorescence were further selected for isolation by quadrant streaking onto PHA detecting agar plates followed by incubation at 37 °C for 24 h [82].

### 3.6. Submerged Fermentation for PHA Production

The PHA positive colonies after isolation were then subjected to submerged fermentation. Seed culture (24 h old) was aseptically inoculated in 50 mL of autoclaved fermentation medium and incubated at 37 °C/150 rpm in shaking incubator for 72 h [64]. The experiment for each sample was run in duplicates.

### 3.7. Extraction of PHA Produced during Fermentation

After 72 h, the PHA content was extracted by sodium hypochlorite and chloroform digestion method of Kumar [71] with a few modifications. The procedure consisted of multiple rounds of centrifugation at 6000 rpm/15 min, drying, and suspension of weighed dried pellets in solutions of 4% sodium hypochlorite and chloroform. It was later proceeded by incubation at 37 °C/150 rpm in a shaking water bath (Daigger Scientific Inc., Wisd, Model: WSB-30) for 1 h. Finally, 1:1 solution of acetone and ethanol was added and pellets were dried in the thermal oven at 60 °C until the liquid content was evaporated. The extracted PHA was collected by filtration on pre-weighed filter paper followed by drying at 60 °C until the achievement of constant weight [83].

### 3.8. Quantification of Produced PHA

Extracted PHA was quantified as percentage production (%PHA) in cell dry weight (CDW). PHA content was determined as a ratio between the total dry weight of extracted PHA to CDW [84].

The following formulas were applied for the quantification [49]:

Cell dry weight (CDW) = weight of falcon tube with dried pellets − weight of empty falcon tube

Dry weight of extracted PHA = weight of filter paper with dried filtered PHA − weight of empty filter paper

$$\%\mathrm{PHA}\;=\;\frac{\;\mathrm{Dry}\;\mathrm{weight}\;\mathrm{of}\;\mathrm{extracted}\;\mathrm{PHA}}{\mathrm{CDW}}\times 100$$

### 3.9. Characterization of the Extracted PHA by FTIR

The PHA extracted from the most efficient PHA producing microbial isolate was sent for Fourier-Transform Infrared spectroscopy (FTIR) (model: IR-Prestige) characterization to Center for Advanced Studied in Physics (CASP), Government College University, Lahore for functional group analysis through signal peaks recorded in the form of percentage transmittance in the range of 4000–400 cm^−1^ [85].

### 3.10. Molecular Identification of the Most Efficient PHA Producing Strain

The most efficient PHA producing bacterial strain was identified by 16S rRNA sequencing by sending samples to Macrogen sequencing company, Seoul, Korea. The sequence of the bacterial strain was further subjected to homology analysis through Basic Local Alignment Search Tool (BLAST) [36]. The phylogenetic tree of the identified bacterial isolate was created using Jalview based upon the BLAST results [86]. After the BLAST, the sequence of the identified strain was submitted into the GenBank nucleotide sequence database via BankIt (MN240936) [87].

### 3.11. Optimization of Cultural Conditions

Four parameters of cultural conditions were optimized for PHA production. The first three parameters included the time of incubation (24–96 h), incubation temperature (25–40 °C), and the pH of the fermentation medium (6.0–8.0). Waste, such as wood chips, cardboard, plastic bottles, wasted polystyrene cups, and waste plastic bags were used as alternate carbon sources instead of glucose in the fermentation medium. Optimization of these sources was done to analyze their potential to be used as cheap, sustainable, and alternate carbon sources [11].

### 3.12. PHA Film Preparation

PHA film was prepared by adding about 5 mL of chloroform in 0.5 g of extracted dried PHA in a 15 mL falcon tube and set for evaporation in a drying oven at 60 °C until chloroform was completely evaporated [30].

### 3.13. Statistical Analysis

Computer software CoStat, cs6204W.exe application was applied to carry out the statistical analysis [88]. All of the experiments were run in duplicates to determine the standard error margins in the final yields. Replicates significant differences were presented as Duncan’s multiple range tests in the form of probability (*p*) values. These calculations were done to validate the reproducibility of the experiments.

## 4. Conclusions

Paint industry soil resulted in the isolation of indigenous and unique PHA producing bacteria *Stenotrophomonas maltophilia* HA-16 (MN240936). Fermentation cultural parameters, such as incubation time, pH, temperature, and carbon sources were found to have a significant effect on PHA production as they increased the PHA yield by 1.16 folds. Moreover, utilizing an untreated plastic bag as a carbon source instead of glucose for PHA production was a standout finding with a yield difference of less than 1.1 folds. However, as the research on this unique strain is extremely limited, it still requires extensive studies to turn this bacteria beneficial for industrial use.

## Figures and Tables

**Table 1:** Geographical distribution of samples used for the isolation of polyhydroxyalkanoates (PHAs) producing bacteria and the number of positive and negative isolates per sample.

Label ^1^	Sample Type	Area in Punjab, Pakistan	Global Positioning System Location ^2^	No. of Isolates	PHA Accumulation ^3^
1	Compost	Kakrali, Gujrat	32°51′8.9352″ N 74°4′25.8132″ E	2	+ in all
2	Soil	Crop field, Mandi-bahauddin	32°32′48.1″ N 73°28′00.4″ E	3	+ in isolates 2a, 2c
3	Effluent	Akzo Nobel Pakistan, paint industry, Lahore	31°28′38.9″ N 74°20′32.1″ E	1	+ in all
4	Soil	Flying Paper industry, Lahore	31°31′47.4″ N 74°21′57.3″ E	1	+ in all
5	Soil	Toyo Nasic Plastic industry, Kot Abdullah, Gujranwala	32.1112° N 74.1967° E	2	+ in 5a, − in 5b
6	Effluent	Royal industries, Pvt. Ltd. Gujranwala	32°08′03.5″ N 74°10′01.9″ E	2	− In all
7	Soil	A field in Narowal city	32°06′28.0″ N 74°52′39.2″ E	3	+ in 7b, 7c
8	Soil	Asia poultry farm, Phool Nagar, Dina Nath	31°12′30.7″ N 73°59′43.2″ E	3	+ in 8b
9	Effluent	Universal Food Industries, Lahore	31°34′58.2″ N 74°18′18.8″ E	3	+in 9c
10	Soil	Corn field, Sargodha	32°02′58.5″ N 72°38′52.5″ E	1	− in all
11	Soil	Shahtaj Sugar Mills, Mandi-Bahauddin	32°35′19.8″ N 73°27′17.1″ E	1	+ in all
12	Soil	Gabol Chowk, Seetpur, Muzaffargarh	29°14′50.3″ N 70°50′25.0″ E	3	+ in all
13	Compost	Kot Shahan, Gujranwala	32°13′51.2″ N 74°10′45.5″ E	2	+ in 13c
14	Soil	Plastic bag landfill site in Mandi-Bahauddin	32°34′41.2″ N 73°29′26.4″ E	2	+ in all
15	Soil	Indus Rice Mills, Mandii-Bahauddin	32°35′51.3″ N 73°29′27.7″ E	2	− in all
16	Soil	Engi Plastic Industries, Lahore	31°19′21.8″ N 74°13′42.4″ E	2	+ in all
17	Effluent	Toyo Nasic Plastic industry, Kot Abdullah, Gujranwala	32.1112° N 74.1967° E	1	+ in all
18	Soil	Incineration plant, Wah cantt, Rawalpindi	33°46′47.8″ N 72°41′05.1″ E	3	+ in 18a, 18b
19	Effluent	Sewage stream in Gujranwala	32°11′58.4″ N 74°10′38.3″ E	2	+ in all
20	Soil	Jadeed plastic industry, Pirwadhai, Rawalpindi	33°38′08.8″ N 73°02′05.2″ E	2	+ in 20a, − in 20b
21	Soil	Fazal Paper Mills (Pvt.) Ltd., Okara	30°52′29.8″ N 73°25′09.6″ E	2	+ in all
22	Effluent	Griffon Plastic Industries (Pvt) Ltd., Kot Lakhpat, Lahore	31°27′02.7″ N 74°20′49.8″ E	1	− in all
23	Soil	Lokhadair Landfill, Lahore	31°37′37.2″ N 74°25′09.0″ E	3	+ in all
24	Soil	Wheat crops in Phalia, Mandi-Bahauddin	32°25′44.7″ N 73°35′27.5″ E	2	− in all
25	Effluent	Z.A. Food Industries, Faisalabad	31°26′25.1″ N 73°01′31.0″ E	3	+ in 25a
26	Effluent	Campbell Flour Mills, Attock	33°45′30.7″ N 72°21′43.6″ E	3	− in all
27	Soil	Gujrat Road, Phalia, Mandi-Bahauddin	32°26′08.8” N 73°36′59.3” E	2	+ in all
28	Soil	Agbro Poultry Breeding Farms, Jajja-Dhok, Rawalpindi	33°19′42.2″ N 73°02′14.0″ E	1	− in all
29	Compost	Near Narowal railway station, Narowal	32°06′02.2″ N 74°51′38.3″ E	1	+ in all
30	Soil	Margalla Packages Industry Plastic Bags Manufacturers, Rawalpindi	33°37′00.1″ N 73°03′09.2″ E	3	+ in all
31	Effluent	Century Paper and Board Mills Ltd., Lahore	31°30′10.3″ N 74°19′30.6″ E	3	+ in 31a, 31c
32	Effluent	Sewage stream, Narowal	32°06′29.9″ N 74°51′43.1″ E	3	− in all
33	Soil	Prime Tanning Industries (Pvt.) Ltd., Sheikhupura	31°46′53.5″ N 74°15′31.7″ E	2	+ in 33a
34	Compost	Alipur, Sheikhupura	31°43′36.0″ N 74°13′38.8″ E	3	+ in 34a, 34c
35	Soil	MashaAllah Poultry Farm, Chak Beli Khan, Rawalpindi	33°16′21.1″ N 72°54′34.7″ E	2	− in all
36	Effluent	Quick Food Industries Mon Salwa, Lahore	31°17′04.0″ N 74°10′42.2″ E	3	+ in 36a, 36b
37	Effluent	Hassan marbles industry, Jehlum	32°55′30.9″ N 73°43′09.5″ E	2	− in all
38	Soil	Lucky Plastic Industries (Pvt) Ltd., Raiwind road, Lahore	31°16′26.3″ N 74°05′47.2″ E	3	+ in all
39	Soil	Noon Sugar Mills Ltd., Bhalwal Road, Sargodha	32°16′44.4″ N 72°55′02.1″ E	1	+ in all
40	Soil	Sewage stream in Kasur	31°06′41.1″ N 74°28′26.4″ E	1	+ in all
41	Compost	Sargodha	32°03′55.6″ N 72°37′58.4″ E	2	+ in all
42	Effluent	Askari Cement Ltd., Wah	33°49′01.3″ N 72°43′25.5″ E	2	− in all
43	Effluent	Jauharabad Sugar Mills Limited, Khushab	32°18′21.7″ N 72°16′28.5″ E	1	+ in all
44	Soil	Wheat field, Sargodha	32°05′38.2″ N 72°42′49.3″ E	1	− in all
45	Soil	Andrew Paints, Islamabad	33°32′35.6″ N 73°08′21.3″ E	2	+ in 45a, − in 45b
46	Soil	Arsam Pulp and Paper Industries Limited, Sheikhupura	31°41′15.8″ N 74°02′26.1″ E	3	+ in 46b, 46c
47	Soil	Amir Food Industry, Faisalabad	31°26′53.7″ N 73°05′50.8″ E	2	+ in all
48	Soil	Hero Plastic Industries, Lahore	31°36′27.1″ N 74°21′14.7″ E	1	+ in all
49	Compost	Sheikhupura	31°43′46.5″ N 74°00′33.7″ E	1	+ in all
50	Soil	Capital Chemical Industries, Rawalpindi	33°30′47.9″ N 73°13′52.7″ E	2	+ in 50a, − in 50b
51	Effluent	Pakistan Paint Factory, Rawalpindi	33°38′50.0″ N 73°03′37.1″ E	2	+ in all
52	Soil	Dandot Cement Factory, Jehlum	32°38′39.4″ N 72°58′34.7″ E	3	+ in 52b
53	Effluent	Dump site, Sheikhupura	31°43′47.5″ N 74°00′02.0″ E	2	+ in all
54	Soil	Kamalia Sugar Mills, Toba Tek Singh	30°45′59.7″ N 72°34′49.5″ E	3	+ in 54a
55	Soil	Oval Ground, Government College University, Lahore	31°34′22.2″ N 74°18′24.8″ E	1	− in all
56	Effluent	Anarkali Bazar, Lahore	31°34′04.7″ N 74°18′16.5″ E	2	− in all
57	Soil	GCU Girls Hostel, Lahore	31°34′08.1″ N 74°18′16.6″ E	1	+ in all
58	Soil	Tariq Plastic Industry, Gujranwala	32°12′12.7″ N 74°10′22.9″ E	1	- in all
59	Effluent	Expert Advertising and Packaging Ltd., Lahore	31°26′29.0″ N 74°19′05.2″ E	2	+ in 59a, − in 59b
60	Soil	Gujranwala	32°06′37.7″ N 74°12′35.7″ E	3	+ in 60c
61	Soil	Pioneer Cement Ltd., Lahore	31°31′29.6″ N 74°19′27.4″ E	2	− in all
62	Soil	Qazi Town, Gujranwala	32°06′41.2″ N 74°12′02.0″ E	2	+ in 62a, − in 62b
63	Soil	Glow Paints Factory, Rawalpindi	33°37′22.5″ N 73°05′46.1″ E	2	− in all
64	Soil	Corn Field, Gujranwala	32°08′02.0″ N 74°13′30.7″ E	2	− in all
65	Soil	Al-Aziz Plastic Industry, Gujranwala	32°11′37.9″ N 74°09′38.5″ E	2	+ in 65a, − in 65b

^1^ Labeling scheme is carried out by numbering the sample and giving each isolate a small alphabetical notation with the number. For example, if Sample 1 has five isolates, they will be named as 1a, 1b, 1c, 1d, and 1e. ^2^ Coordinates of some locations are approximate. ^3^ PHA accumulation is determined by the presence of orange fluorescence when illuminated under UV at 315 nm: + for PHA presence, − for PHA absence.

**Table 2:** Percentage PHA accumulation by isolated strains after submerged fermentation with glucose as a carbon source.

Sample Label (No. of Isolates with +PHA Production)	Isolate	Average PHA Production of the Isolate (%PHA)	Sample Label (No. of Isolates with +PHA Production)	Isolate	Average PHA Production of the Isolate (%PHA)
1 (2)	1a	19.93 ± 0.25	33 (1)	33a	38.22 ± 0.24
	1b	16.15 ± 0.19	34 (2)	34a	17.25 ± 0.13
				34c	12.02 ± 0.28
2 (2)	2a	35.66 ± 0.16	36 (2)	36a	25.22 ± 0.22
				36b	21.36 ± 0.15
	2c	29.27 ± 0.48	38 (3)	38a	31.25 ± 0.36
				38b	26.12 ± 0.23
				38c	29.55 ± 0.37
3 (1)	3a	43.76 ± 0.18	39 (1)	39a	36.25 ± 0.51
4 (1)	4a	37.59 ± 0.22	40 (1)	40a	45.23 ± 0.23
5 (1)	5a	41.2 ± 0.18	41 (2)	41a	15.96 ± 0.29
				41b	17.69 ± 0.35
7 (1)	7a	15.22 ± 0.28	43 (1)	43a	34.51 ± 0.21
8 (1)	8b	52.78 ± 0.23	45 (1)	45a	38.22 ± 0.45
9 (1)	9c	22.98 ± 0.50	46 (2)	46b	30.55 ± 0.31
				46c	28.63 ± 0.26
11 (1)	11a	39.68 ± 0.21	47 (2)	47a	39.21 ± 0.38
				47b	35.25 ± 0.48
12 (3)	12a	37.00 ± 0.15	48 (1)	48a	53.24 ± 0.15
	12b	8.87 ± 0.17			
	12c	2.09 ± 0.18			
13 (1)	13c	19.37 ± 0.39	49 (1)	49a	21.03 ± 0.14
14 (2)	14a	43.75 ± 0.35	50 (1)	50a	15.96 ± 0.23
	14b	16.79 ± 0.46			
16 (2)	16a	69.72 ± 0.17	51 (2)	51a	11.23 ± 0.53
	16b	55.98 ± 0.19		51b	13.24 ± 0.27
17 (1)	17a	24.33 ± 0.21	52 (1)	52b	8.03 ± 0.19
18 (2)	18a	8.64 ± 0.34	53 (2)	53a	36.66 ± 0.16
	18b	38.97 ± 0.27		53b	31.26 ± 0.22
19 (2)	19a	11.56 ± 0.42	54 (1)	54a	17.36 ± 0.18
	19b	27.67 ± 0.18			
20 (1)	20a	35.92 ± 0.31	57 (1)	57a	20.36 ± 0.35
21 (2)	21a	27.83 ± 0.29	59 (1)	59a	33.29 ± 0.12
	21b	28.07 ± 0.23			
23 (3)	23a	48.68 ± 0.46	60 (1)	60c	25.25 ± 0.35
	23b	26.88 ± 0.28			
	23c	15.68 ± 0.39			
25 (1)	25a	12.77 ± 0.38	62 (1)	62a	29.25 ± 0.11
27 (2)	27a	6.87 ± 0.40	65 (1)	65a	38.15 ± 0.41
	27b	12.68 ± 0.20			
29 (1)	29a	15.68 ± 0.39			
30 (3)	30a	30.36 ± 0.27			
	30b	25.99 ± 0.19			
	30c	29.11 ± 0.23			
31 (2)	31a	19.00 ± 0.11			
	31c	18.90 ± 0.28			

**Table 3:** Primers used for PCR as well as 16S rRNA sequencing.

PCR Primers	Sequencing Primers
27F 5′ (AGA GTT TGA TCM TGG CTC AG) 3′	785F 5′ (GGA TTA GAT ACC CTG GTA) 3′
1492R 5′ (TAC GGY TAC CTT GTT ACG ACT T) 3′	907R 5′ (CCG TCA ATT CMT TTR AGT TT) 3′

**Table 4:** Comparison of maximum PHA produced during optimization of various cultural conditions.

Parameter	Optimized at	Average PHA Production (%PHA)	Intracellular CDW (g/100 mL)
Incubation time	24 h	30.02 ± 0.27	0.02
	48 h	39.64 ± 0.16	0.07
	72 h	65.39 ± 0.42	0.52
	96 h	47.56 ± 0.37	0.38
Temperature of incubation	25 °C	41.81 ± 0.39	0.06
	30 °C	47.32 ± 0.12	0.07
	37 °C	78.85 ± 0.23	0.59
	40 °C	30.41 ± 0.47	0.28
pH of the fermentation medium	6.0	20.14 ± 0.26	0.05
	6.5	41.70 ± 0.29	0.11
	7.0	78.85 ± 0.11	0.19
	7.5	32.95 ± 0.33	0.07
	8.0	30.09 ± 0.18	0.07
Carbon source	Glucose (standard)	78.08 ± 0.19	0.50
	Plastic bag	68.24 ± 0.27	0.19
	Wood chips	53.15 ± 0.17	0.16
	Cardboard cutouts	51.76 ± 0.48	0.15
	Waste shredded polystyrene cups	43.75 ± 0.30	0.17
	Plastic bottle cutouts	38.19 ± 0.22	0.14
